# Supplementary material for: Assessing pyrethroid resistance status in the Culex pipiens complex (Diptera: Culicidae) from the northwest suburbs of Chicago, Illinois using Cox regression of bottle bioassays and other detection tools
Source: PLoS One. 2022 Jun 29;17(6):e0268205. doi: 10.1371/journal.pone.0268205 (PMC9242439; doi:10.1371/journal.pone.0268205)
Supplement: S1 File — (PDF) [file pone.0268205.s001.pdf]

Supplement 1

Edwin R. Burgess IV, Kristina Lopez, Patrick Irwin, Collin P. Jaeger, and Alden S. Estep

Assessing Pyrethroid Resistance Status in the *Culex pipiens* Complex (Diptera: Culicidae) from the Northwest Suburbs of Chicago, Illinois Using Cox Regression of Bottle Bioassays and Other Detection Tools

Load the necessary libraries

```
library(tidyverse)
library(survival)
```

Read the data into R

```
CxBottle = read.csv('Cx_Chicago_Bottle_2022.csv')

# Quick check of the data (rows, columns, data types, etc.)
glimpse(CxBottle)
```

```
## Rows: 315
## Columns: 7
## $ Dead    <int> 0, 1, 7, 13, 14, 16, 19, 19, 20, 20, 20, 20, 20, 20, 23, 0, 5,~
## $ Trmt    <chr> "Sumithrin", "Sumithrin", "Sumithrin", "Sumithrin", "Sumithrin~
## $ Min     <int> 5, 10, 15, 20, 25, 30, 35, 40, 45, 60, 75, 90, 105, 120, 120, ~
## $ Status  <int> 1, 1, 1, 1, 1, 1, 1, 1, 1, 1, 1, 1, 1, 1, 0, 1, 1, 1, 1, 1, 1,~
## $ Strain  <chr> "AHB", "AHB", "AHB", "AHB", "AHB", "AHB", "AHB", "AHB", "AHB",~
## $ Date    <chr> "Aug_5", "Aug_5", "Aug_5", "Aug_5", "Aug_5", "Aug_5", "Aug_5",~
## $ Cluster <chr> "B01", "B01", "B01", "B01", "B01", "B01", "B01", "B01", "B01",~
```

```
head(CxBottle, 20)
```

```
##    Dead      Trmt Min Status Strain  Date Cluster
## 1     0 Sumithrin   5      1    AHB Aug_5     B01
## 2     1 Sumithrin  10      1    AHB Aug_5     B01
## 3     7 Sumithrin  15      1    AHB Aug_5     B01
## 4    13 Sumithrin  20      1    AHB Aug_5     B01
## 5    14 Sumithrin  25      1    AHB Aug_5     B01
## 6    16 Sumithrin  30      1    AHB Aug_5     B01
## 7    19 Sumithrin  35      1    AHB Aug_5     B01
## 8    19 Sumithrin  40      1    AHB Aug_5     B01
## 9    20 Sumithrin  45      1    AHB Aug_5     B01
## 10   20 Sumithrin  60      1    AHB Aug_5     B01
## 11   20 Sumithrin  75      1    AHB Aug_5     B01
## 12   20 Sumithrin  90      1    AHB Aug_5     B01
## 13   20 Sumithrin 105      1    AHB Aug_5     B01
## 14   20 Sumithrin 120      1    AHB Aug_5     B01
```

```
## 15   23 Sumithrin 120      0    AHB Aug_5     B01
## 16    0 Sumithrin   5      1    AHB Aug_5     B02
## 17    5 Sumithrin  10      1    AHB Aug_5     B02
## 18   13 Sumithrin  15      1    AHB Aug_5     B02
## 19   13 Sumithrin  20      1    AHB Aug_5     B02
## 20   16 Sumithrin  25      1    AHB Aug_5     B02
```

```
# Create an 'adjusted dead' column that changes cumulative deaths
# into new deaths at each time step.
CxBottle$AdjDead = sapply(1:nrow(CxBottle),
                          function(x)
                            if (x == 1 || any(x == which(CxBottle$Status==0)+1))
                            {CxBottle[x,1]}
                          else {CxBottle[x,1] - CxBottle[x-1,1]}
                          )
```

```
# Create a 'survtrim' function to allow selection of start time (t1) and stop time (t2)
survtrim = function(n = 1, t1 = 0, df.in, sp, t2) {

  l = split(df.in, df.in[, sp])

  df.keep = l[[n]] %>% filter(t1 <= Min, Min <= t2)

  df.sum = l[[n]] %>% filter(Min > t2)

  df.add = df.sum %>% filter(Status == 0)

  df.add$Min = t2

  df.add$AdjDead = sum(df.sum$AdjDead)

  df.out = rbind(df.keep, df.add) %>%
    distinct()

  df.out

}
```

```
# Determine the number of 'clusters' from the data
nclust = length(unique(CxBottle$Cluster))

# Use the 'survtrim' function on each 'cluster' in the data
list.out = lapply(1 : nclust,
                  survtrim,
                  df.in = CxBottle,
                  sp = 'Cluster',
                  t1 = 0, # change starting time
                  t2 = 30) # change end time

# Combine the output from a list into a single dataframe
df.done = bind_rows(list.out)
```

```
head(df.done, 35)
```

```
##    Dead      Trmt Min Status Strain  Date Cluster AdjDead
## 1     0 Sumithrin   5      1    AHB Aug_5     B01       0
## 2     1 Sumithrin  10      1    AHB Aug_5     B01       1
## 3     7 Sumithrin  15      1    AHB Aug_5     B01       6
## 4    13 Sumithrin  20      1    AHB Aug_5     B01       6
## 5    14 Sumithrin  25      1    AHB Aug_5     B01       1
## 6    16 Sumithrin  30      1    AHB Aug_5     B01       2
## 7    23 Sumithrin  30      0    AHB Aug_5     B01       7
## 8     0 Sumithrin   5      1    AHB Aug_5     B02       0
## 9     5 Sumithrin  10      1    AHB Aug_5     B02       5
## 10   13 Sumithrin  15      1    AHB Aug_5     B02       8
## 11   13 Sumithrin  20      1    AHB Aug_5     B02       0
## 12   16 Sumithrin  25      1    AHB Aug_5     B02       3
## 13   18 Sumithrin  30      1    AHB Aug_5     B02       2
## 14   24 Sumithrin  30      0    AHB Aug_5     B02       6
## 15    1 Sumithrin   5      1    AHB Aug_5     B03       1
## 16    2 Sumithrin  10      1    AHB Aug_5     B03       1
## 17    6 Sumithrin  15      1    AHB Aug_5     B03       4
## 18    9 Sumithrin  20      1    AHB Aug_5     B03       3
## 19   14 Sumithrin  25      1    AHB Aug_5     B03       5
## 20   15 Sumithrin  30      1    AHB Aug_5     B03       1
## 21   23 Sumithrin  30      0    AHB Aug_5     B03       8
## 22    2     Anvil   5      1    AHB Aug_7     B11       2
## 23    5     Anvil  10      1    AHB Aug_7     B11       3
## 24   14     Anvil  15      1    AHB Aug_7     B11       9
## 25   22     Anvil  20      1    AHB Aug_7     B11       8
## 26   23     Anvil  25      1    AHB Aug_7     B11       1
## 27   23     Anvil  30      1    AHB Aug_7     B11       0
## 28   23     Anvil  30      0    AHB Aug_7     B11       0
## 29    7     Anvil   5      1    AHB Aug_7     B12       7
## 30   17     Anvil  10      1    AHB Aug_7     B12      10
## 31   20     Anvil  15      1    AHB Aug_7     B12       3
## 32   20     Anvil  20      1    AHB Aug_7     B12       0
## 33   20     Anvil  25      1    AHB Aug_7     B12       0
## 34   20     Anvil  30      1    AHB Aug_7     B12       0
## 35   20     Anvil  30      0    AHB Aug_7     B12       0
```

Statistical Analysis

```
data30 = df.done %>%
  uncount(weights = AdjDead) %>%
  dplyr::select(-Dead)
```

Refer to Therneau (2021) for further inquiries about clustered Cox regression and time-dependent covariates.

AHB

Subset the data with the two treatments you want to compare. Here is the Sumithrin treatment from Aug 5 and the Anvil treatment from Aug 7.

```
AHBSurv = subset(data30,
                 Strain == "AHB" & Date == "Aug_5" |
                   Strain == "AHB" & Date == "Aug_7")
```

Build the Cox regression model. "Cluster" represents each bottle run during the tests. Here there are three bottles of Sumithrin and three bottles of Anvil.

```
AHBFit = coxph(Surv(Min, Status) ~ factor(Trmt == "Anvil") + cluster(Cluster),
               data=AHBSurv)
```

Assessing proportional hazard across the observed time. In this simple setup, the global and treatment df and P-value will be equal because there is only one factor in the model. A significant P-value implies that hazard is not proportional across time and a time-dependent coefficient is needed in the model. Here proportional hazard can be assumed.

```
cox.zph(AHBFit)
```

```
##                         chisq df    p
## factor(Trmt == "Anvil") 0.618  1 0.43
## GLOBAL                  0.618  1 0.43
```

The assumption of proportional hazards is met since the P-value of cox.zph() is greater than or equal to 0.05. No time-dependent coefficients are necessary here.

Plot of the proportional hazard test (Schoenfeld residuals).

```
plot(cox.zph(AHBFit))

abline(0,0, col = 2)

abline(h = AHBFit$coef[1],
       col = 3,
       lwd = 2,
       lty = 2)
```

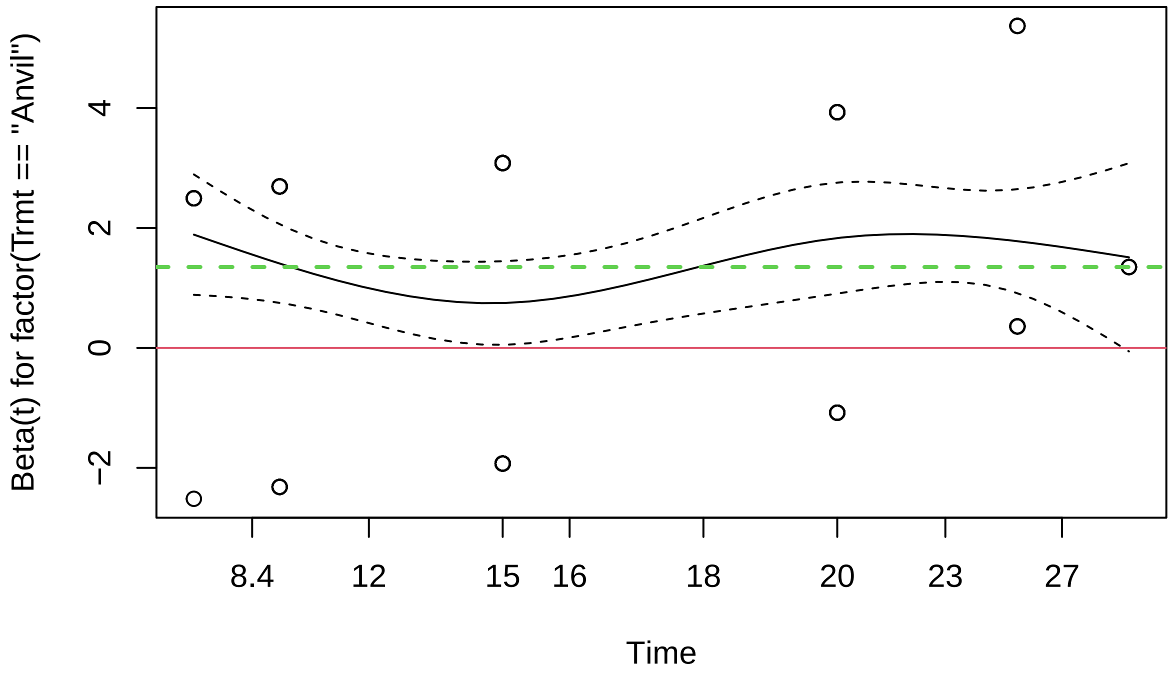

```r
summary(AHBFit)
```

```
## Call:
## coxph(formula = Surv(Min, Status) ~ factor(Trmt == "Anvil"),
##     data = AHBSurv, cluster = Cluster)
##
##   n= 135, number of events= 114
##
##                               coef exp(coef) se(coef) robust se     z Pr(>|z|)
## factor(Trmt == "Anvil")TRUE 1.3491    3.8539   0.2097    0.2215 6.089 1.13e-09
##
## factor(Trmt == "Anvil")TRUE ***
## ---
## Signif. codes:  0 '***' 0.001 '**' 0.01 '*' 0.05 '.' 0.1 ' ' 1
##
##                             exp(coef) exp(-coef) lower .95 upper .95
## factor(Trmt == "Anvil")TRUE     3.854     0.2595     2.496     5.949
##
## Concordance= 0.676  (se = 0.036 )
## Likelihood ratio test= 43.08  on 1 df,   p=5e-11
## Wald test            = 37.08  on 1 df,   p=1e-09
## Score (logrank) test = 46.67  on 1 df,   p=8e-12,   Robust = 4.95  p=0.03
##
##   (Note: the likelihood ratio and score tests assume independence of
##      observations within a cluster, the Wald and robust score tests do not).
```

There is a significant difference in the hazard rate of mortality between Anvil and Sumithrin (p = 1.13e-09).

The hazard ratio is 3.854, which indicates that there is a 3.854-fold change in the hazard rate of mortality of the Anvil treatment whe compared to the Sumithrin treatment.

WHE

Sumithrin vs. Anvil (0-30 min)

```
WHESumAnvSurv=subset(data30,Strain=="WHE"&Date=="Aug_11"|Strain=="WHE"&Date=="Aug_14")
```

Build the Cox regression model. "Cluster" represents each bottle run during the tests. Here there are six bottles of Sumithrin and seven bottles of Anvil.

```
WHESumAnvFit=coxph(Surv(Min,Status)~factor(Trmt=="Anvil")+cluster(Cluster),
                   data=WHESumAnvSurv)
```

Assessing proportional hazard across the observed time. In this simple setup, the global and treatment df and P-value will be equal because there is only one factor in the model. A significant P-value implies that hazard is not proportional across time and a time-dependent coefficient is needed in the model.

```
cox.zph(WHESumAnvFit)
```

```
##                          chisq df      p
## factor(Trmt == "Anvil")   9.96  1 0.0016
## GLOBAL                    9.96  1 0.0016
```

Here proportional hazard is not assumed and a time-dependent coefficient will need to be added.

Plot of the proportional hazard test (Shoenfeld residuals).

```
plot(cox.zph(WHESumAnvFit))

abline(0,0,col=2)

abline(h=WHESumAnvFit$coef[1],col=3,lwd=2,lty=2)
```

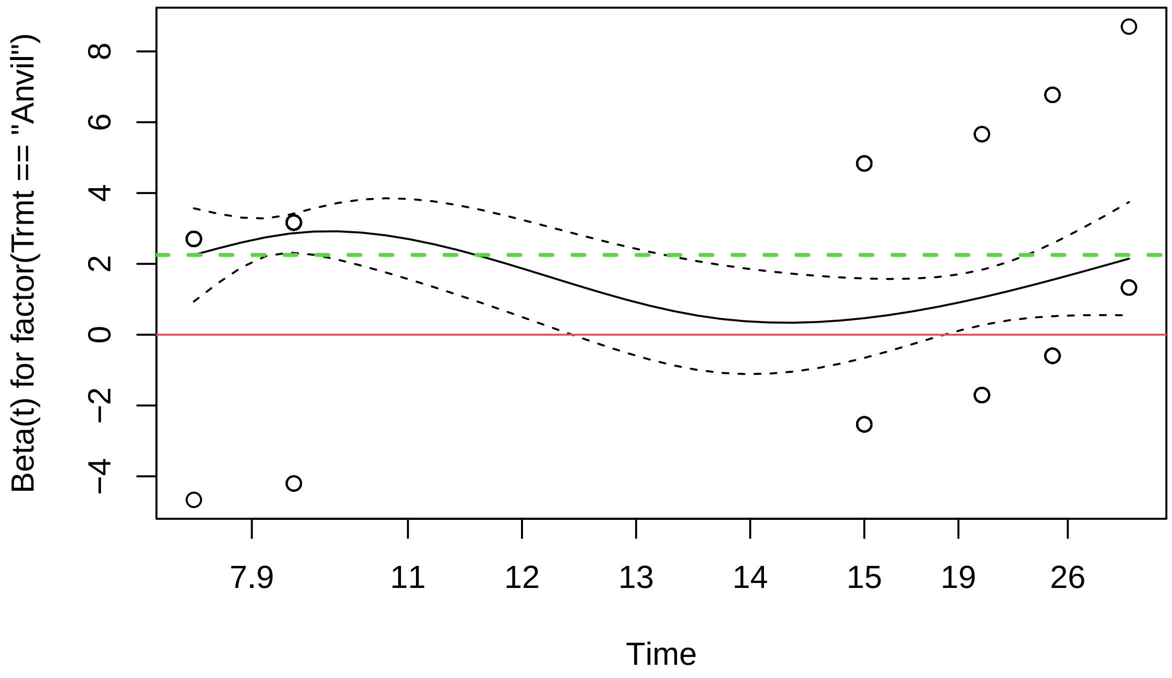

Hazard rate changes with time among the treatments. Thus, an interaction-like term is applied x*t using the tt function.

```
WHESumAnvFittt=coxph(Surv(Min,Status)~factor(Trmt=="Anvil")+
                       tt(Trmt=="Anvil")+cluster(Cluster),
                     data=WHESumAnvSurv,tt=function(x,t,...)x*t)
```

```
summary(WHESumAnvFittt)
```

```
## Call:
## coxph(formula = Surv(Min, Status) ~ factor(Trmt == "Anvil") +
##     tt(Trmt == "Anvil"), data = WHESumAnvSurv, tt = function(x,
##     t, ...) x * t, cluster = Cluster)
##
##   n= 200, number of events= 164
##
##                                 coef exp(coef) se(coef) robust se      z
## factor(Trmt == "Anvil")TRUE  3.58646  36.10604  0.67690   0.74008  4.846
## tt(Trmt == "Anvil")         -0.09050   0.91347  0.04282   0.04228 -2.141
##                             Pr(>|z|)
## factor(Trmt == "Anvil")TRUE 1.26e-06 ***
## tt(Trmt == "Anvil")           0.0323 *
## ---
## Signif. codes:  0 '***' 0.001 '**' 0.01 '*' 0.05 '.' 0.1 ' ' 1
##
##                             exp(coef) exp(-coef) lower .95 upper .95
## factor(Trmt == "Anvil")TRUE   36.1060     0.0277    8.4649  154.0069
## tt(Trmt == "Anvil")            0.9135     1.0947    0.8408    0.9924
##
## Concordance= 0.788  (se = 0.026 )
## Likelihood ratio test= 145.7  on 2 df,   p=<2e-16
## Wald test            = 506.8  on 2 df,   p=<2e-16
## Score (logrank) test = 143.1  on 2 df,   p=<2e-16,   Robust = 8.74  p=0.01
##
##   (Note: the likelihood ratio and score tests assume independence of
##      observations within a cluster, the Wald and robust score tests do not).
```

There is a significant difference between the hazard rate of mortality in the Anvil treatment compared to the Sumithrin treatment. The hazard ratio was not proportional over time by a factor of 0.91. The hazard ratio at time zero was 36.1. This value changes for each time point by a factor of 0.91^n (where n is equal to the time step in minutes). Thus, the equation to calculate hazard rate of mortality at any given time step is: 36.1 * 0.91^n.

Sumithrin vs. AnvilTM (0-30 min)

```
WHESumvAnvTSurv=subset(data30,Strain=="WHE"&Date=="Aug_11"|Strain=="WHE"&Date=="Aug_13")

WHESumvAnvTFit=coxph(Surv(Min,Status)~factor(Trmt=="AnvilTM")+cluster(Cluster),
                     data=WHESumvAnvTSurv)

cox.zph(WHESumvAnvTFit)
```

```
##                            chisq df       p
## factor(Trmt == "AnvilTM")  10.9  1 0.00097
## GLOBAL                     10.9  1 0.00097
```

Plot of the proportional hazard test (Shoenfeld residuals).

```
plot(cox.zph(WHESumvAnvTFit))

abline(0,0,col=2)

abline(h=WHESumvAnvTFit$coef[1],col=3,lwd=2,lty=2)
```

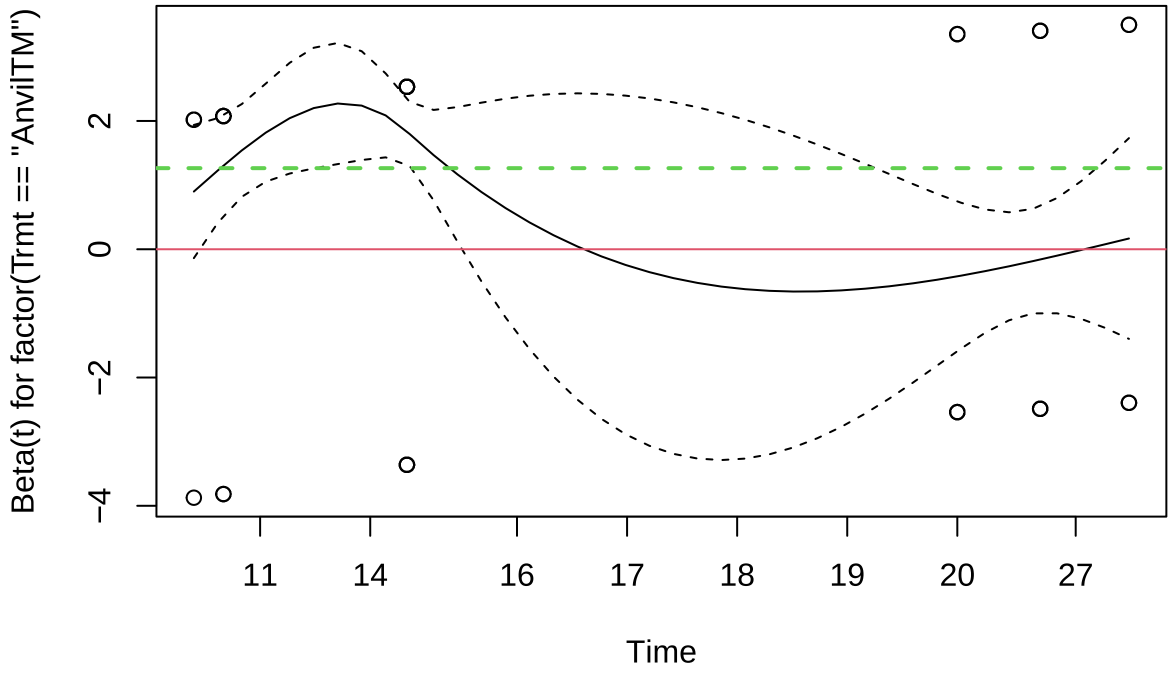

Hazard rate changes with time among the treatments, thus an interaction-like term is applied x*t using the tt function.

```
WHESumvAnvTFit=coxph(Surv(Min,Status)~factor(Trmt=="AnvilTM")+
                       tt(Trmt=="AnvilTM")+cluster(Cluster),
                     data=WHESumvAnvTSurv,tt=function(x,t,...)x*t)
```

```
summary(WHESumvAnvTFit)
```

```
## Call:
## coxph(formula = Surv(Min, Status) ~ factor(Trmt == "AnvilTM") +
##     tt(Trmt == "AnvilTM"), data = WHESumvAnvTSurv, tt = function(x,
##     t, ...) x * t, cluster = Cluster)
##
##   n= 217, number of events= 165
##
##                                   coef exp(coef) se(coef) robust se      z
## factor(Trmt == "AnvilTM")TRUE  2.85254  17.33168  0.68841   0.66505  4.289
## tt(Trmt == "AnvilTM")         -0.09184   0.91225  0.03737   0.03139 -2.926
##                               Pr(>|z|)
## factor(Trmt == "AnvilTM")TRUE 1.79e-05 ***
## tt(Trmt == "AnvilTM")          0.00344 **
## ---
## Signif. codes:  0 '***' 0.001 '**' 0.01 '*' 0.05 '.' 0.1 ' ' 1
##
##                               exp(coef) exp(-coef) lower .95 upper .95
## factor(Trmt == "AnvilTM")TRUE   17.3317     0.0577    4.7070   63.8167
## tt(Trmt == "AnvilTM")            0.9122     1.0962    0.8578    0.9701
##
## Concordance= 0.679  (se = 0.041 )
## Likelihood ratio test= 59.37  on 2 df,   p=1e-13
## Wald test            = 75.4  on 2 df,   p=<2e-16
## Score (logrank) test = 52.3  on 2 df,   p=4e-12,   Robust = 7.19  p=0.03
##
##   (Note: the likelihood ratio and score tests assume independence of
##      observations within a cluster, the Wald and robust score tests do not).
```

There is a significant difference between the hazard rate of mortality in the Anvil treatment compared to the Sumithrin treatment. The hazard ratio was not proportional over time by a factor of 0.91. The hazard ratio at time zero was 17.3. This value changes for each time point by a factor of 0.91^n (where n is equal to the time step in minutes). Thus, the equation to calculate hazard rate of mortality at any given time step is: 17.3 * 0.91^n.

Works Cited:

Therneau T. M. 2021. https://cran.r-project.org/web/packages/survival/survival.pdf
