# Supplementary material for: Assessing pyrethroid resistance status in the Culex pipiens complex (Diptera: Culicidae) from the northwest suburbs of Chicago, Illinois using Cox regression of bottle bioassays and other detection tools
Source: PLoS One. 2022 Jun 29;17(6):e0268205. doi: 10.1371/journal.pone.0268205 (PMC9242439; doi:10.1371/journal.pone.0268205)
Supplement: Attachment — Submitted filename: Response to Reviewers.docx [file pone.0268205.s003.docx]

**Response to Reviewers:**

**Response to Reviewer #1:**
Methods
In the section “Mosquito sources and pyrethroid exposure histories” the authors should add information on common aquatic habitats for Culex larvae in the different study sites.

*****We appreciate this comment, but we did not do thorough surveillance of the aquatic habitats that *Culex* were developing in, so we would only be hazarding a guess if we added this information here. We do note that mosquitoes were collected “in residential neighborhoods with numerous parks, schools, and manicured lawns.” Based on this description, all common *Culex* larval habitats in residential neighborhoods would be possible.**

Line 123 to 128 “Sprays were conducted once a week for 5 weeks in Wheeling, Arlington Heights North, and Des Plaines South in 2019 and 2020, starting in July and ending in August. In 2019, Zenivex® E20 (20% etofenprox) in a 1:1 mix with mineral oil (10% etofenprox) was used and in 2020, Anvil® 10+10 was sprayed at 0.0036 Lb per acre of active ingredient and piperonyl butoxide (PBO). From 2013 – 2018 Northwest Mosquito Abatement District averaged 1 spray event (etofenprox) per year in these areas.”
What area is sprayed? is it houses? Water collection? What??? Please add information.

*****Thank you for this comment. A reference to Fig. 1, the map of the study area, was added to line 129 to clarify which areas were treated. We added additional information on the adulticide applications on line 132-133 – “Truck-mounted ULV adulticide sprays were applied with a London Fog 18-20 (London Foggers, Minneapolis, MN) to all drivable roads within each area.”**

Results
The authors say they did mosquito identification in the methodology but no information on the identity of species recorded by sites is presented.

*****We appreciate this comment, but the study organism was *Culex pipiens* and we did not quantify or characterize any of the other (few) species of *Culex* collected.**

The authors also say haven used both male and females for bioassay but no information on the susceptibility of each group to insecticides is provided. I will propose that they add information on males and females susceptibility to insecticide. This data could improve comprehension and interpretation of their findings.

*****Thank you for this comment. We did not differentiate between males and females in the bottle bioassays, nor were they differentiated for enzyme activity or *kdr* testing. CONUS guidelines suggest using both males and females together (Section 3.1. of the following link:** [**https://www.cdc.gov/mosquitoes/pdfs/conus-508.pdf**](https://www.cdc.gov/mosquitoes/pdfs/conus-508.pdf)**) Also, please see Question 6 in the Appendix on Page 18.**

Genotyping for knockdown resistance alleles
The authors should include details of mosquito processed were these mosquitoes survivors to bioassays? were they dead? or mosquitoes not exposed?

*****Clarified at line 159. Now says: “Individual organisms collected from the treated bottles of the CDC bottle bioassays were loaded into 96-well plates (Omni International, Kennesaw, GA) with 400 µL of nuclease free water and cubic zirconium beads (BioSpec Products, Bartlesville, OK).”**

Enzyme activity assays
The authors should add the precision whether the expression profile reported is for resistant samples or for samples not exposed to insecticide.

*****This is stated at lines 196-199: “The content of mixed-function oxidases (MFO), and the activity of α- and β-carboxylesterases (α-, β-CarE), and glutathione *S*-transferases (GST) were determined using modified methods from [22] using only freeze-killed control mosquitoes from the bottle bioassays (i.e., mosquitoes that were not exposed to any toxicant).”**

**Response to Reviewer #2:**

**Comment 1 (line 31): We have added the species authority (we assume the reviewer meant authority and not attribution) as well as the Order and Family.**

**Comment 2 (line 54): We have added a citation here**

**Comment 3 (line 71): Change made to “relatively less expensive.”**

**Comment 4 (line 75): We have chosen to take out “a few” and replace it with “certain.” This avoids a relatively meaningless magnitude statement (i.e., “few” or “several”)**

**Comment 5 (line 83): Added reference to Fig 1 here as requested.**

**Comment 6 (line 88): We feel that cutting these last two sentences and moving them to the discussion loses a lot of context that is required for the sentence above it to make sense. This reviewer felt that we explained the Cox regression component well in the discussion so we do not feel that moving it adds anything to the discussion that isn’t already there.**

**Comment 7 (line 118): To avoid this confusion we provide both the real location as well as the canonical location of the kdr mutation in the manuscript. We have added “(canonical L1014F)” here to add further clarity.**

**Comment 8 (line 127): We disagree that these last four sentences should be moved to the introduction. Reviewer #1 wanted us to add some more context about where sprays were done here and doing so would give this section more important methodological information that we feel should stay in the methods section.**

**Comment 9 (line 149): Added a statement “At least two replicates of each bottle were run, for each location and insecticide solution (Table 1).” Table 1 has been modified to have the number of replicates per insecticide solution at each location.**

**Comment 10 (line 153): We have addressed this comment (Comment 7 above).**

**Comment 11 (line 156): We have added the following: “LL” was defined as homozygous wildtype, “FF” homozygous *kdr*, and “LF” heterozygous.”**

**Comment 12 (line 168): Thank you for this comment. Yes, the assay works fine with just homogenate. Melt curve kdr assay from homogenate has been described previously for *Aedes aegypti* by both Audrey Lenhardt from CDC using “squish buffer” and Estep using nuclease free water. Notably, to the comment, homogenization in smaller volumes than we use here (400ul) tends to inhibit PCR.**

**Comment 13 (line 175): The number tested per location are included in Table 2 but in all cases were a minimum of 37 individuals. We have added: “A minimum of 37 individual organisms were tested for each location (Table 2).”**

**Comment 14 (line 188): Thank you for the kind comment but we stand on the shoulders of previous work. Credit to Saavedra-Rodriguez [22] for being the source of adding the tails to increase the Tm difference between the various SNPs. We just adapted the assays from *Aedes aegypti* to be specific for *Culex quinquefasciatus*.**

**Comment 15 (line 323): The text has been edited to be more descriptive and clear.**

**Comment 16 (line 330): The text has been updated to define the results of the controls. “Results of the included genotype control samples in this melt curve assay matched previously conducted Sanger sequencing on the same samples.”**

**Comment 17 (line 352): Thank you for this comment. We would prefer to keep it as “some” because we did not surveil all of the northwest suburbs of Chicago, so it is technically more accurate.**

**Comment 18 (line 381): We have clarified the definitions of the numbering system earlier in the manuscript now.**

**Comment 19 (line 382): We appreciate the comment but we are unclear what the reviewer is referring to when saying “just hangs here.” We have tried to make an effort to clarify by adding the following: “In the case of the *Cx. pipiens* complex, hybridization rate among its congeners may also play a role in expression of *kdr* [32].”**

**Comment 20 (line 397): Changed “close relative” to “congener.”**

**Comment 21 (line 419): Thank you for this kind comment.**

**Comment 22 (line 476): Thank you for this kind comment as well.**

**Response to Editor:**

When submitting your revision, we need you to address these additional requirements.

1. Please ensure that your manuscript meets PLOS ONE's style requirements, including those for file naming. The PLOS ONE style templates can be found at 
[https://journals.plos.org/plosone/s/file?id=wjVg/PLOSOne_formatting_sample_main_body.pdf](https://urldefense.proofpoint.com/v2/url?u=https-3A__journals.plos.org_plosone_s_file-3Fid-3DwjVg_PLOSOne-5Fformatting-5Fsample-5Fmain-5Fbody.pdf&d=DwMFAg&c=sJ6xIWYx-zLMB3EPkvcnVg&r=hLlaY5LoPcK_v13ePfg0FWFmOWzi9P8zNZ1ONch8VwY&m=RISSLF3bKNlqtonfH-noIO_ti6c2xc1X6UExsOoQGfRGT0SLivSyPUo_7fGHPojn&s=OzkLSyblw06bC9w74wQ0vGVfxfFEIvefzx3ZxrrOygw&e=) and 
[https://journals.plos.org/plosone/s/file?id=ba62/PLOSOne_formatting_sample_title_authors_affiliations.pdf](https://urldefense.proofpoint.com/v2/url?u=https-3A__journals.plos.org_plosone_s_file-3Fid-3Dba62_PLOSOne-5Fformatting-5Fsample-5Ftitle-5Fauthors-5Faffiliations.pdf&d=DwMFAg&c=sJ6xIWYx-zLMB3EPkvcnVg&r=hLlaY5LoPcK_v13ePfg0FWFmOWzi9P8zNZ1ONch8VwY&m=RISSLF3bKNlqtonfH-noIO_ti6c2xc1X6UExsOoQGfRGT0SLivSyPUo_7fGHPojn&s=iL-WPrcPe99dQeR49f56UDRtpkdC3zNuGcZlm6pfByI&e=)

2. In your Methods section, please provide additional information regarding the permits you obtained for the work. Please ensure you have included the full name of the authority that approved the field site access and, if no permits were required, a brief statement explaining why.

*****Permits were not required to work at homes because homeowners gave verbal permission to use their property. This is stated in the manuscript. The only permit needed for this was an NPDES general permit for pesticide application point source discharges. The permit number was ILG870138 for NWMAD. This has been added to the manuscript.**

3. We note that Figure 1 in your submission contain [map/satellite] images which may be copyrighted. All PLOS content is published under the Creative Commons Attribution License (CC BY 4.0), which means that the manuscript, images, and Supporting Information files will be freely available online, and any third party is permitted to access, download, copy, distribute, and use these materials in any way, even commercially, with proper attribution. For these reasons, we cannot publish previously copyrighted maps or satellite images created using proprietary data, such as Google software (Google Maps, Street View, and Earth). For more information, see our copyright guidelines: [http://journals.plos.org/plosone/s/licenses-and-copyright](https://urldefense.proofpoint.com/v2/url?u=http-3A__journals.plos.org_plosone_s_licenses-2Dand-2Dcopyright&d=DwMFAg&c=sJ6xIWYx-zLMB3EPkvcnVg&r=hLlaY5LoPcK_v13ePfg0FWFmOWzi9P8zNZ1ONch8VwY&m=RISSLF3bKNlqtonfH-noIO_ti6c2xc1X6UExsOoQGfRGT0SLivSyPUo_7fGHPojn&s=yAQrhdeAj7Cvpb906CeTb4ZQq5GPWPQz33eiLQW4-iM&e=).
 
We require you to either (1) present written permission from the copyright holder to publish these figures specifically under the CC BY 4.0 license, or (2) remove the figures from your submission:
 
a. You may seek permission from the original copyright holder of Figure 1 to publish the content specifically under the CC BY 4.0 license.  
 
We recommend that you contact the original copyright holder with the Content Permission Form ([http://journals.plos.org/plosone/s/file?id=7c09/content-permission-form.pdf](https://urldefense.proofpoint.com/v2/url?u=http-3A__journals.plos.org_plosone_s_file-3Fid-3D7c09_content-2Dpermission-2Dform.pdf&d=DwMFAg&c=sJ6xIWYx-zLMB3EPkvcnVg&r=hLlaY5LoPcK_v13ePfg0FWFmOWzi9P8zNZ1ONch8VwY&m=RISSLF3bKNlqtonfH-noIO_ti6c2xc1X6UExsOoQGfRGT0SLivSyPUo_7fGHPojn&s=ZhTpJMNmITScdjdwuMasaC8a31bDzRu-v8SguXFyxhg&e=)) and the following text:
“I request permission for the open-access journal PLOS ONE to publish XXX under the Creative Commons Attribution License (CCAL) CC BY 4.0 ([http://creativecommons.org/licenses/by/4.0/](https://urldefense.proofpoint.com/v2/url?u=http-3A__creativecommons.org_licenses_by_4.0_&d=DwMFAg&c=sJ6xIWYx-zLMB3EPkvcnVg&r=hLlaY5LoPcK_v13ePfg0FWFmOWzi9P8zNZ1ONch8VwY&m=RISSLF3bKNlqtonfH-noIO_ti6c2xc1X6UExsOoQGfRGT0SLivSyPUo_7fGHPojn&s=CpoutpoZkPQIoOJLBMwM3N3tl50kZXUU3Ykpd-L0Hv8&e=)). Please be aware that this license allows unrestricted use and distribution, even commercially, by third parties. Please reply and provide explicit written permission to publish XXX under a CC BY license and complete the attached form.”
 
Please upload the completed Content Permission Form or other proof of granted permissions as an ""Other"" file with your submission.
 
In the figure caption of the copyrighted figure, please include the following text: “Reprinted from [ref] under a CC BY license, with permission from [name of publisher], original copyright [original copyright year].”
 
b. If you are unable to obtain permission from the original copyright holder to publish these figures under the CC BY 4.0 license or if the copyright holder’s requirements are incompatible with the CC BY 4.0 license, please either i) remove the figure or ii) supply a replacement figure that complies with the CC BY 4.0 license. Please check copyright information on all replacement figures and update the figure caption with source information. If applicable, please specify in the figure caption text when a figure is similar but not identical to the original image and is therefore for illustrative purposes only.
The following resources for replacing copyrighted map figures may be helpful:
 
USGS National Map Viewer (public domain): [http://viewer.nationalmap.gov/viewer/](https://urldefense.proofpoint.com/v2/url?u=http-3A__viewer.nationalmap.gov_viewer_&d=DwMFAg&c=sJ6xIWYx-zLMB3EPkvcnVg&r=hLlaY5LoPcK_v13ePfg0FWFmOWzi9P8zNZ1ONch8VwY&m=RISSLF3bKNlqtonfH-noIO_ti6c2xc1X6UExsOoQGfRGT0SLivSyPUo_7fGHPojn&s=I6fnGJ2omjZ3F7Y3tjJ2dld2qhnfT_vOjH9693NN2F4&e=)
The Gateway to Astronaut Photography of Earth (public domain): [http://eol.jsc.nasa.gov/sseop/clickmap/](https://urldefense.proofpoint.com/v2/url?u=http-3A__eol.jsc.nasa.gov_sseop_clickmap_&d=DwMFAg&c=sJ6xIWYx-zLMB3EPkvcnVg&r=hLlaY5LoPcK_v13ePfg0FWFmOWzi9P8zNZ1ONch8VwY&m=RISSLF3bKNlqtonfH-noIO_ti6c2xc1X6UExsOoQGfRGT0SLivSyPUo_7fGHPojn&s=KH-TvS_-bCJSBaLVR8VZ0KydGtGSQPat4gtcSIlNLIw&e=)
Maps at the CIA (public domain): [https://www.cia.gov/library/publications/the-world-factbook/index.html](https://urldefense.proofpoint.com/v2/url?u=https-3A__www.cia.gov_library_publications_the-2Dworld-2Dfactbook_index.html&d=DwMFAg&c=sJ6xIWYx-zLMB3EPkvcnVg&r=hLlaY5LoPcK_v13ePfg0FWFmOWzi9P8zNZ1ONch8VwY&m=RISSLF3bKNlqtonfH-noIO_ti6c2xc1X6UExsOoQGfRGT0SLivSyPUo_7fGHPojn&s=iVrNI7F4DWvruSxPrc4SgGgx7R7DAEd-rWkp1NeIb74&e=)and [https://www.cia.gov/library/publications/cia-maps-publications/index.html](https://urldefense.proofpoint.com/v2/url?u=https-3A__www.cia.gov_library_publications_cia-2Dmaps-2Dpublications_index.html&d=DwMFAg&c=sJ6xIWYx-zLMB3EPkvcnVg&r=hLlaY5LoPcK_v13ePfg0FWFmOWzi9P8zNZ1ONch8VwY&m=RISSLF3bKNlqtonfH-noIO_ti6c2xc1X6UExsOoQGfRGT0SLivSyPUo_7fGHPojn&s=-UOmnJ0OXbO8IHRAVSASdwaIgTm3eufHnC4dIYrj9C0&e=)
NASA Earth Observatory (public domain): [http://earthobservatory.nasa.gov/](https://urldefense.proofpoint.com/v2/url?u=http-3A__earthobservatory.nasa.gov_&d=DwMFAg&c=sJ6xIWYx-zLMB3EPkvcnVg&r=hLlaY5LoPcK_v13ePfg0FWFmOWzi9P8zNZ1ONch8VwY&m=RISSLF3bKNlqtonfH-noIO_ti6c2xc1X6UExsOoQGfRGT0SLivSyPUo_7fGHPojn&s=GNxFzM8GyJ4T3yBykZfR_GHWe4B8SLZyUnkrUrN8EkM&e=)
Landsat: [http://landsat.visibleearth.nasa.gov/](https://urldefense.proofpoint.com/v2/url?u=http-3A__landsat.visibleearth.nasa.gov_&d=DwMFAg&c=sJ6xIWYx-zLMB3EPkvcnVg&r=hLlaY5LoPcK_v13ePfg0FWFmOWzi9P8zNZ1ONch8VwY&m=RISSLF3bKNlqtonfH-noIO_ti6c2xc1X6UExsOoQGfRGT0SLivSyPUo_7fGHPojn&s=xPt9cuYcW3fOTU8DzY4WpKjfibLKeinzSTgKn0AGo1Q&e=)
USGS EROS (Earth Resources Observatory and Science (EROS) Center) (public domain): [http://eros.usgs.gov/#](https://urldefense.proofpoint.com/v2/url?u=http-3A__eros.usgs.gov_-23&d=DwMFAg&c=sJ6xIWYx-zLMB3EPkvcnVg&r=hLlaY5LoPcK_v13ePfg0FWFmOWzi9P8zNZ1ONch8VwY&m=RISSLF3bKNlqtonfH-noIO_ti6c2xc1X6UExsOoQGfRGT0SLivSyPUo_7fGHPojn&s=dhni68vNiauxN9ZHrqacKSFbo_4817Z8rGPDLkVy43A&e=)
Natural Earth (public domain): [http://www.naturalearthdata.com/](https://urldefense.proofpoint.com/v2/url?u=http-3A__www.naturalearthdata.com_&d=DwMFAg&c=sJ6xIWYx-zLMB3EPkvcnVg&r=hLlaY5LoPcK_v13ePfg0FWFmOWzi9P8zNZ1ONch8VwY&m=RISSLF3bKNlqtonfH-noIO_ti6c2xc1X6UExsOoQGfRGT0SLivSyPUo_7fGHPojn&s=S7iUIovY_f5mHLJUpt0YpG_37EBePjflG9ogrryOzkg&e=)

*****We thank you for this attention. Fig. 1 was made by co-author Patrick Irwin using Esri ArcMap 10.8. The Cook County and Chicago layer are publicly available at** [**https://hub-cookcountyil.opendata.arcgis.com/**](https://hub-cookcountyil.opendata.arcgis.com/)**. If this does not suffice for permissions, we will gladly fill out the permission forms upon request**

4. Please review your reference list to ensure that it is complete and correct. If you have cited papers that have been retracted, please include the rationale for doing so in the manuscript text, or remove these references and replace them with relevant current references. Any changes to the reference list should be mentioned in the rebuttal letter that accompanies your revised manuscript. If you need to cite a retracted article, indicate the article’s retracted status in the References list and also include a citation and full reference for the retraction notice.

*****References are complete and accurate. We did not identify any retracted citations.**
